# Enzymatic hydrolysis of waste streams originating from wastewater treatment plants

**DOI:** 10.1186/s13068-024-02553-x

**Published:** 2024-07-18

**Authors:** Ruta Zarina, Linda Mezule

**Affiliations:** Water Systems and Biotechnology Institute, Faculty of Natural Sciences and Technology, Riga Technical University, Kipsalas Iela 6a, Riga, Latvia

**Keywords:** Sewage sludge, Enzymatic hydrolysis, Resource recovery, Sugar production, Waste treatment

## Abstract

**Background:**

Achieving climate neutrality is a goal that calls for action in all sectors. The requirements for improving waste management and reducing carbon emissions from the energy sector present an opportunity for wastewater treatment plants (WWTPs) to introduce sustainable waste treatment practices. A common biotechnological approach for waste valorization is the production of sugars from lignocellulosic waste biomass via biological hydrolysis. WWTPs produce waste streams such as sewage sludge and screenings which have not yet been fully explored as feedstocks for sugar production yet are promising because of their carbohydrate content and the lack of lignin structures. This study aims to explore the enzymatic hydrolysis of various waste streams originating from WWTPs by using a laboratory-made and a commercial cellulolytic enzyme cocktail for the production of sugars. Additionally, the impact of lipid and protein recovery from sewage sludge prior to the hydrolysis was assessed.

**Results:**

Treatment with a laboratory-made enzyme cocktail produced by *Irpex lacteus* (IL) produced 31.2 mg sugar per g dry wastewater screenings. A commercial enzyme formulation released 101 mg sugar per g dry screenings, corresponding to 90% degree of saccharification. There was an increase in sugar levels for all sewage substrates during the hydrolysis with IL enzyme. Lipid and protein recovery from primary and secondary sludge prior to the hydrolysis with IL enzyme was not advantageous in terms of sugar production.

**Conclusions:**

The laboratory-made fungal IL enzyme showed its versatility and possible application beyond the typical lignocellulosic biomass. Wastewater screenings are well suited for valorization through sugar production by enzymatic hydrolysis. Saccharification of screenings represents a viable strategy to divert this waste stream from landfill and achieve the waste treatment and renewable energy targets set by the European Union. The investigation of lipid and protein recovery from sewage sludge showed the challenges of integrating resource recovery and saccharification processes.

## Background

In the era of circular economy, waste management in the European Union (EU) follows the waste hierarchy and requires approaches such as reuse, recycling and recovery of resources. Based on this hierarchy, the least preferred option is waste disposal in landfills due to the loss of resources. The waste policy in the EU is committed to restricting the landfilling of any waste suitable for recycling or energy recovery after 2030 [1]. Furthermore, the energy sector in the EU is aiming to increase the share of renewable fuels to 42.5% by 2030 to reduce the greenhouse gas emissions [2]. These activities are a part of the larger EU goal of achieving climate neutrality by 2050 [3].

The application of resource-efficient technologies is particularly crucial in such an indispensable industry as wastewater treatment [4]. Owing to the increasing effluent quality standards, the efforts to remove pollution from the aquatic environment are increasingly more energy-intensive [5]. Wastewater treatment plants (WWTPs) produce not just treated effluent but also various kinds of waste streams, including sewage sludge, screenings and sewage grit. The management of sludge is a well-known area of research, and the conventional treatment methods include composting and anaerobic digestion. Over the years, resource recovery from sewage sludge has become more popular and is now a growing trend in sludge management [6]. Meanwhile, the valorization of screenings and sewage grit is less explored. The most common fate of screenings and sewage grit currently is landfilling [7, 8]. The advantage of applying novel treatment strategies to waste streams originating from WWTPs is that they are separated, rich in organic matter and readily available [9]. Due to the interconnectedness of water, waste and energy sectors, the municipal WWTP is at the centre of circular economy innovations [10].

Screenings are collected from the screening units at the primary treatment stage. They have a heterogeneous composition, including solid materials such as disposable hygiene products, plastics, paper, cardboard, vegetable scraps, and hair [11]. Grit removal is another process in primary wastewater treatment. Sewage grit consists of small suspended particles, including sand, gravel, seeds and eggshells [12]. After the removal of large solids and grit, wastewater enters the primary clarifier allowing the solids to settle. The settled material is known as primary sludge, and it is has a high content of suspended and dissolved organic matter [13]. By far the most common wastewater treatment type is the activated sludge process which utilizes the activity of microorganisms to degrade pollutants. The excess microbial biomass makes up secondary sludge. A common technology for the treatment of primary and secondary sludge is anaerobic digestion which produces biogas as the main product as well as digested sludge as a co-product [14]. Digested sludge has a reduced organic content compared to primary or secondary sludge.

The necessity of waste resource recovery along with the renewable energy targets and the promising composition of wastewater-related feedstocks reveals an opportunity for high value molecule production. The waste streams in WWTPs are expected to contain cellulosic materials such as papers, textiles and plant residues which are suitable for bioethanol production [15]. Generally, there are three steps in bioethanol production: pretreatment (preparation of the biomass), hydrolysis (sugar production) and fermentation (bioethanol production) [16]. The production of sludge hydrolysate and its fermentation to produce bioethanol has been explored [17–21], showing the efforts to link wastewater treatment, waste valorization and bioenergy production.

The goal of pretreatment is enhancing the substrate susceptibility to hydrolysis, which is particularly relevant for lignocellulosic materials where lignin and hemicellulose are hindering the enzyme access to cellulose [22]. Pretreatment may involve steps such as grinding, thermal treatment, acid or alkali addition, and removal of toxic components [23]. However, sewage sludge and other wastes from WWTPs are not expected to contain lignin in a significant quantity because paper products have been processed to remove lignin whereas food waste (plant residues) is managed outside the wastewater treatment system. Lignin content in sewage sludge is reported as 2–5% [24, 25]. Therefore, an extensive pretreatment targeting the lignin structure is not required for sewage sludge. Considering that the complex composition of sewage sludge makes it a candidate for the recovery of resources such as proteins and lipids [26], we propose replacing the conventional pretreatment of primary and secondary sludge with a resource recovery workflow. This strategy intends to utilize the carbohydrate fraction of sludge for sugar production while the remaining sludge resources are recovered and used in other ways. A similar approach has been attempted with sewage sludge by utilizing the lipids for biodiesel production and then using the lipid-extracted sludge for saccharification and bioethanol production [27]. As demonstrated by Supaporn et al. [28], lipid extraction from sewage sludge can be performed without any significant loss of carbohydrates or proteins.

Enzymatic hydrolysis is regarded as a sustainable and efficient approach to saccharification due to its high sugar yields, mild reaction conditions, and lack of inhibitory compound generation [29, 30]. In this type of hydrolysis, the transformation of complex carbohydrates into fermentable sugars is achieved by enzymes. Various microorganisms have developed the capability of enzymatically degrading biomass, including white-rot fungi which are well-known producers of lignocellulolytic enzymes such as cellulases, hemicellulases and laccases [31].

Although lignocellulosic biomass such as plant residues is a very popular feedstock for bioethanol production, some progress has been made with exploring the treatment of waste biomass. Enzymatic hydrolysis with commercial cellulolytic preparations has been performed on primary sludge [32], paper mill sludge [19, 20, 33], chemically enhanced primary sludge [17], secondary sludge [32], digested sludge [32], and screenings [34].

Alternatively, enzyme cocktails can be produced non-commercially from various fungi. *Irpex lacteus*, a white-rot fungus, is one of the most effective biomass decomposers due to its diverse enzymatic profile [35]. Enzyme mixtures produced by *I. lacteus* show very high enzymatic activity [36] and have been applied to lignocellulosic materials such as hay, wheat straw and grass [37, 38]. Other fungal sources of cellulolytic enzymes that have been investigated include *Aspergillus niger* [39, 40], *Trichoderma reesei* [39–41], *Trichoderma harzianum* [40], *Trichoderma viride* [42], *Pleurotus ostreatus* [36] and *Penicillium janthinellum* [43]. The application of non-commercially prepared fungal enzymes to wastewater related feedstocks has not been widely investigated. For example, cellulose recovered by sieving wastewater was hydrolysed by enzymes produced by *Trichoderma harzianum* [44], and paper sludge was treated with cellulases produced by *Talaromyces cellulolyticus* [21].

This study aims to evaluate the potential of fermentable sugar production from various non-agricultural waste substrates via biological hydrolysis. For the first time, a laboratory-made *Irpex lacteus* enzyme preparation was applied to waste streams originating from wastewater treatment plants. The sugar yield and saccharification efficiency was determined and compared to a commercial cellulase preparation under mild treatment conditions. The effect of lipid and protein recovery from primary and secondary sludge has been tested within this study to assess whether pretreatment enables higher sugar yields.

## Methods

### Design of the study

Several waste streams generated in WWTPs were collected, characterized and subjected to laboratory-scale enzymatic hydrolysis to evaluate their potential for sugar production. Resource recovery was tested as a pretreatment strategy for two of the waste samples. All substrates were hydrolysed with a fungal enzyme mixture prepared under laboratory conditions, and a selection of substrates was also hydrolysed with a commercially available cellulase enzyme.

### Samples

Primary sludge, digested sludge, screenings and sewage grit were collected from a WWTP in Latvia (site coordinates 57.0264, 24.0003; population equivalent (PE) 700 000) where the share of industrial wastewater was 15%. Screenings and sewage grit were dried until constant weight, and screenings were additionally ground (*Retsch*, *Grindomix GM 200*) to a smaller size. Secondary sludge was collected from another WWTP in Latvia (56.9330, 23.6582; PE 30 000) where the share of industrial wastewater was 5%. For the production of protein-free sludge, secondary sludge was collected from another WWTP (56.7766, 23.9122; PE 14 000) where the share of industrial wastewater was 47%.

Protein-free sludge was obtained after protein extraction by ultrasound-assisted chemical hydrolysis [45]. The ultrasonic treatment (79 W, 40 kHz) was conducted in an ultrasonic bath for 2 h at 150 rpm stirring, and it had two stages: first in alkali conditions (using 2.8 M sodium hydroxide), followed by acidic conditions (using 3 M sulfuric acid). After each stage, the hydrolysate was centrifuged at 4000 rpm for 20 min to obtain a protein solution and a precipitate. 300 g sludge and 50 mL alkali was used for the first treatment stage, whereas 100 g of the precipitate and 150 mL acid was used for the second stage. The final precipitate was used in this study as protein-free sludge.

Lipid-free sludge was obtained after lipid extraction from primary sludge. The extraction was carried out in a Soxhlet apparatus using hexane as a solvent. 1 g of air-dried primary sludge was weighed, transferred into a thimble and 100 mL hexane was placed in 500 mL round bottom flask equipped with Soxhlet apparatus and a condenser. The extraction was performed at 80 ºC for 6 h. After extraction, the hexane was removed using a rotary evaporator, and then the extracted lipids were dried under vacuum for 24 h. The final dried material was used as lipid-free sludge.

Screenings, sewage grit and lipid-free sludge were stored at room temperature in plastic containers. Primary, secondary, digested and protein-free sludge were stored at –18 °C before experiments.

### Sample characterization

Total solids (TS) content was determined by drying the samples at 105 °C overnight. Volatile and fixed solids content was determined by heating the dried samples at 550 °C for 4 h. Substrate composition was characterized by the content of carbohydrates, proteins and lipids that were determined spectrophotometrically by the phenol–sulfuric acid method [46], Lowry method [47] and sulpho-phospho-vanillin method [48], respectively. The analytical methods followed the procedure described in Zarina and Mezule [26].

### Enzymatic hydrolysis

Enzymatic hydrolysis tests was performed with a commercial cellulolytic enzyme (*Cellic CTec2*, *Sigma Aldrich,* CC enzyme) and a laboratory-made fungal enzyme mixture produced by the white-rot fungus *Irpex lacteus* (IL enzyme) using hay as the carbon source [38]. Enzyme activity was determined by a standard FPU method [49] and expressed as filter paper units (FPU) per mL of produced enzyme. The enzymatic activity of IL enzyme and CC enzyme was 8 FPU/mL and 184 FPU/mL, respectively.

Hydrolysis experiment flasks contained the sample substrate at 2% w/v loading (per dry weight), sodium citrate (0.05 M, pH 4.8, *Sigma Aldrich*) and the enzyme. Enzyme loading was 4 FPU/g dry substrate for both enzymes. After adding the citrate solution to the substrates, the samples were heated for 5 min on a hot plate, followed by cooling down and enzyme addition. Hydrolysis occurred at 30 °C for 48 h in a shaking incubator (150 rpm). The experimental conditions of the enzymatic hydrolysis (temperature, time, enzyme loading) were selected to model a scenario with a low consumption of energy and resources. Sample aliquots for sugar analysis were taken at 0 h (before enzyme addition), 24 h and 48 h, and were stored at –18 °C until analysis. Additionally, a control experiment was performed where the substrates were incubated without the enzymes. All experimental conditions were tested in triplicates.

### Reducing sugar assay

The concentration of reducing sugars was determined by the dinitrosalicylic acid (DNS) method with glucose as the reference compound [50]. Samples were prepared by centrifugation at 10000 rcf for 5 min. The supernatant was used for sugar analysis. Briefly, for the analysis with a spectrophotometer (*Genesys 150, ThermoScientific*), 0.1 mL sample was combined with 0.1 mL sodium citrate (0.05 M) and 0.6 mL DNS reagent, heated for 5 min, followed by adding 4 mL distilled water. For analysis with a microplate reader (*CLARIOStar Plus, BMG Labtech*), the volumes used in the method were scaled down to 10, 10, 60 and 220 µL, respectively. Absorption was measured at 540 nm wavelength. Furthermore, sugar concentration was determined in the enzymatic preparations, and the final results were corrected for the amount of sugars originating from the enzymes. The results of sugar yield were expressed as mg sugars per g dry substrate (mg/g).

The degree of saccharification (DS) refers to the extent of total carbohydrates converted to reducing sugars during the hydrolysis, calculated by Eq. 1 and expressed as a percentage. Additionally, the changes of DS over time were calculated by Eq. 2 and used to describe the increase in sugar yield attributed to the experimental enzyme activity.

1 $${DS}_{48 h}=\frac{Sugar\, yield\, at\, 48 h}{Total\, carbohydrates}$$

2 $$\Delta {DS}_{48 h}=D{S}_{48h}-D{S}_{0h}$$

### Statistical analysis

The results are expressed as the average ± standard deviation. Differences between groups were compared by a t-test in Microsoft Excel and the cut-off value for statistical significance was *p* = 0.05.

## Results

### Substrate characterization

Prior to the hydrolysis, the substrate properties were determined (Table 1). The substrates had a wide range of moisture content due to their different origins and pretreatments. Carbohydrate content ranged from 3.7% for digested sludge to 11.2% for screenings, indicating the potential for sugar production from the latter. Resource recovery was investigated as a pretreatment prior to the enzymatic hydrolysis of sludge. Specifically, proteins were removed from secondary sludge to produce protein-free sludge, whereas lipids were extracted from primary sludge to produce lipid-free sludge. Prior to protein recovery, secondary sludge contained 19.8% proteins but after the recovery 5.3% proteins remained in protein-free sludge. Primary sludge initially contained 9.1% lipids but the lipid-free sludge contained 2.8% lipids. The content of carbohydrates decreased during the preparation of lipid-free sludge (10.0% to 4.2%) while in protein-free sludge it stayed approximately the same (9.7% to 10.7%).

**Table 1:** Substrate characterization

	Total solids, % ww	Volatile solids, % TS	Carbohydrates, % TS	Proteins, % TS	Lipids, % TS
Primary sludge	4.3	75.2	10.0^a^	23.9^a^	9.1^a^
Secondary sludge	15.3	84.2	9.2	19.8	12.1
Lipid-free sludge	97.8	76.5	4.2	5.5	2.8
Protein-free sludge	8.1	76.9	10.7	5.3	8.5
Digested sludge	22.4	65.5	7.5	8.6^a^	8.0^a^
Screenings	91.1	91.7	11.2	0.5	21.7
Sewage grit	88.8	n.d	5.5	n.d	n.d

*TS* total solids, *ww* wet weight, *n.d*. not determined

^a^Zarina and Mezule [26]

### Enzymatic hydrolysis

#### EH with CC enzyme

**Fig. 1:**
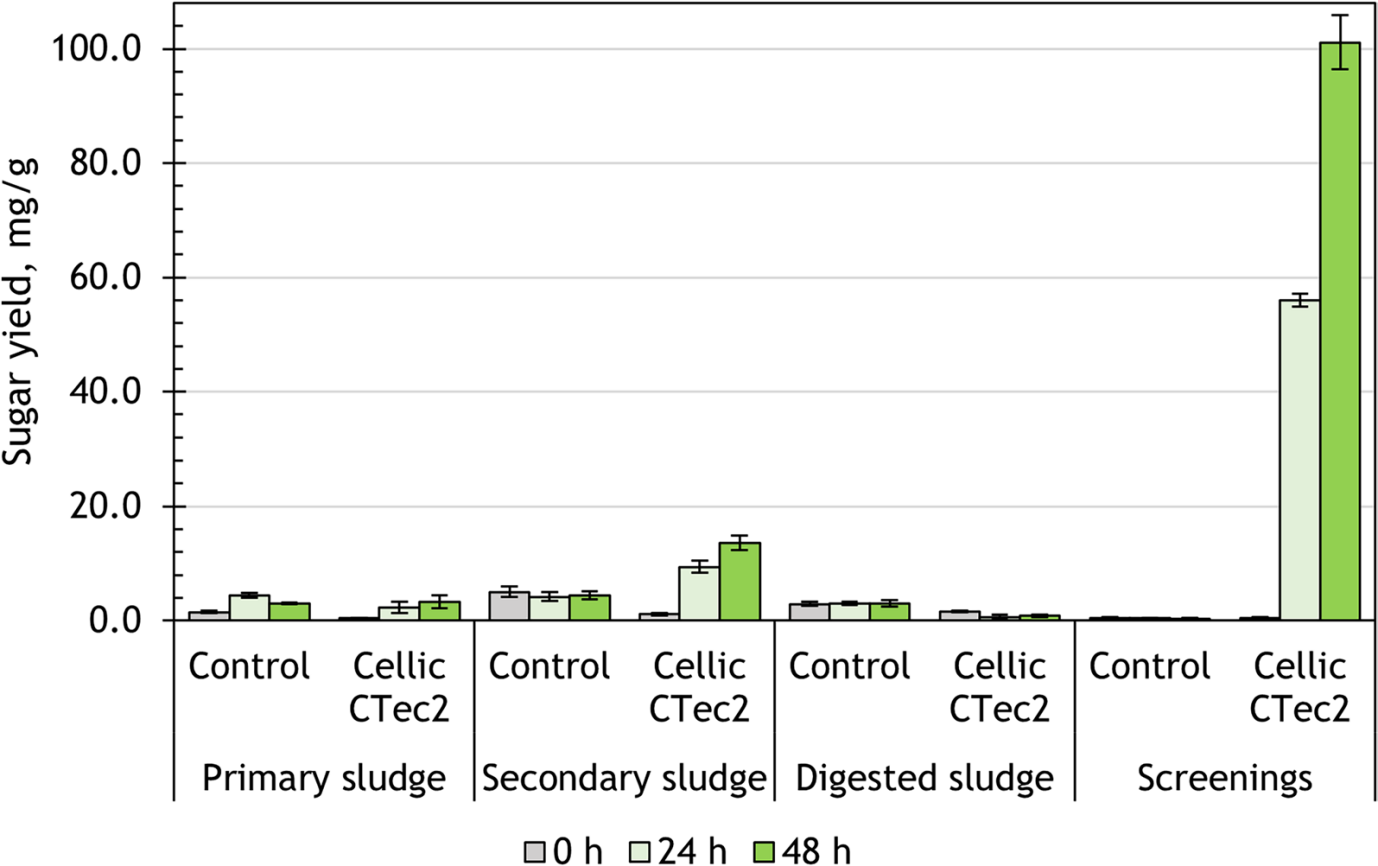
Sugar yield after 48 h of enzymatic hydrolysis with CC enzyme

Primary sludge, secondary sludge, digested sludge and screenings were treated with the commercial CC enzyme. After 48 h of hydrolysis, the sugar yield was 3.3 ± 1.1 mg/g for primary sludge, 13.6 ± 1.3 mg/g for secondary sludge, 0.84 ± 0.05 mg/g for digested sludge and 101.1 ± 12.1 mg/g for screenings (Fig. 1).

**Fig. 2:**
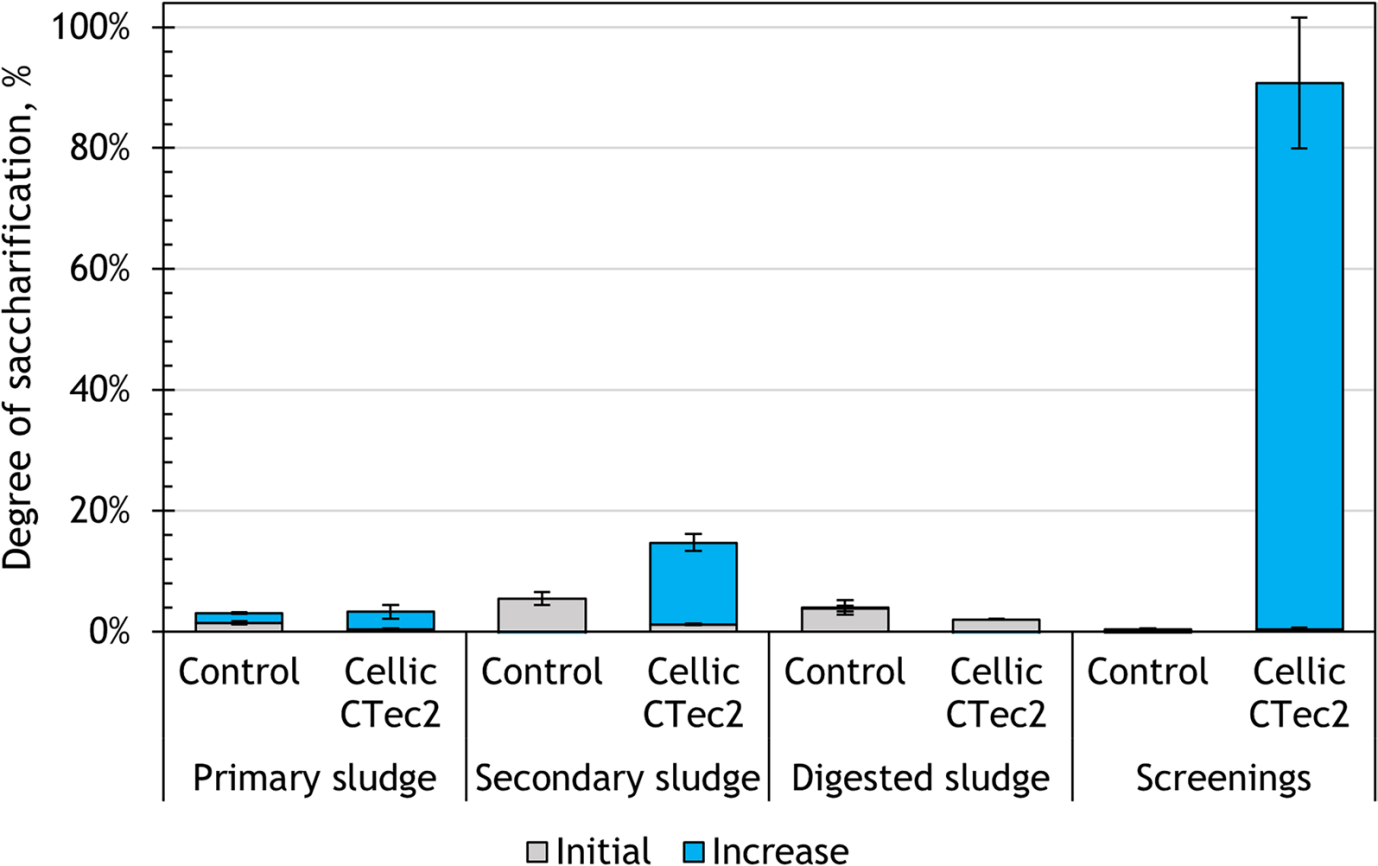
Changes in the degree of saccharification after 48 h of enzymatic hydrolysis with CC enzyme

The initial level of sugars in the hydrolysates was between 0 and 5% (represented by stacked bars in Fig. 2). During the experiment, the degree of saccharification reached 90% for screenings, whereas for secondary sludge it was 15%, for primary sludge—3%, and for digested sludge—1% (Fig. 2). The sugar yield of screenings and secondary sludge increased significantly after 48 h of hydrolysis (*p* < 0.05), compared to the control experiment. In contrast, there were no significant changes in the sugar release from primary sludge, and the amount of sugars released from digested sludge decreased over the course of hydrolysis. Looking at the changes over time, the sugar yield of screenings increased linearly, and could theoretically reach 100% saccharification at 52 h of hydrolysis. Sugar release from secondary sludge was not significantly different at 24 and 48 h, indicating that the process had stabilized at 24 h.

The results show that the selected hydrolysis conditions (such as temperature, time, enzyme loading) using CC enzyme were not compatible with the use of primary and digested sludge. The sugar release from secondary sludge using CC enzyme was unremarkable. In contrast, screenings responded very well to the treatment with CC enzyme, reaching a near complete saccharification of the carbohydrate fraction.

#### EH with IL enzyme

**Fig. 3:**
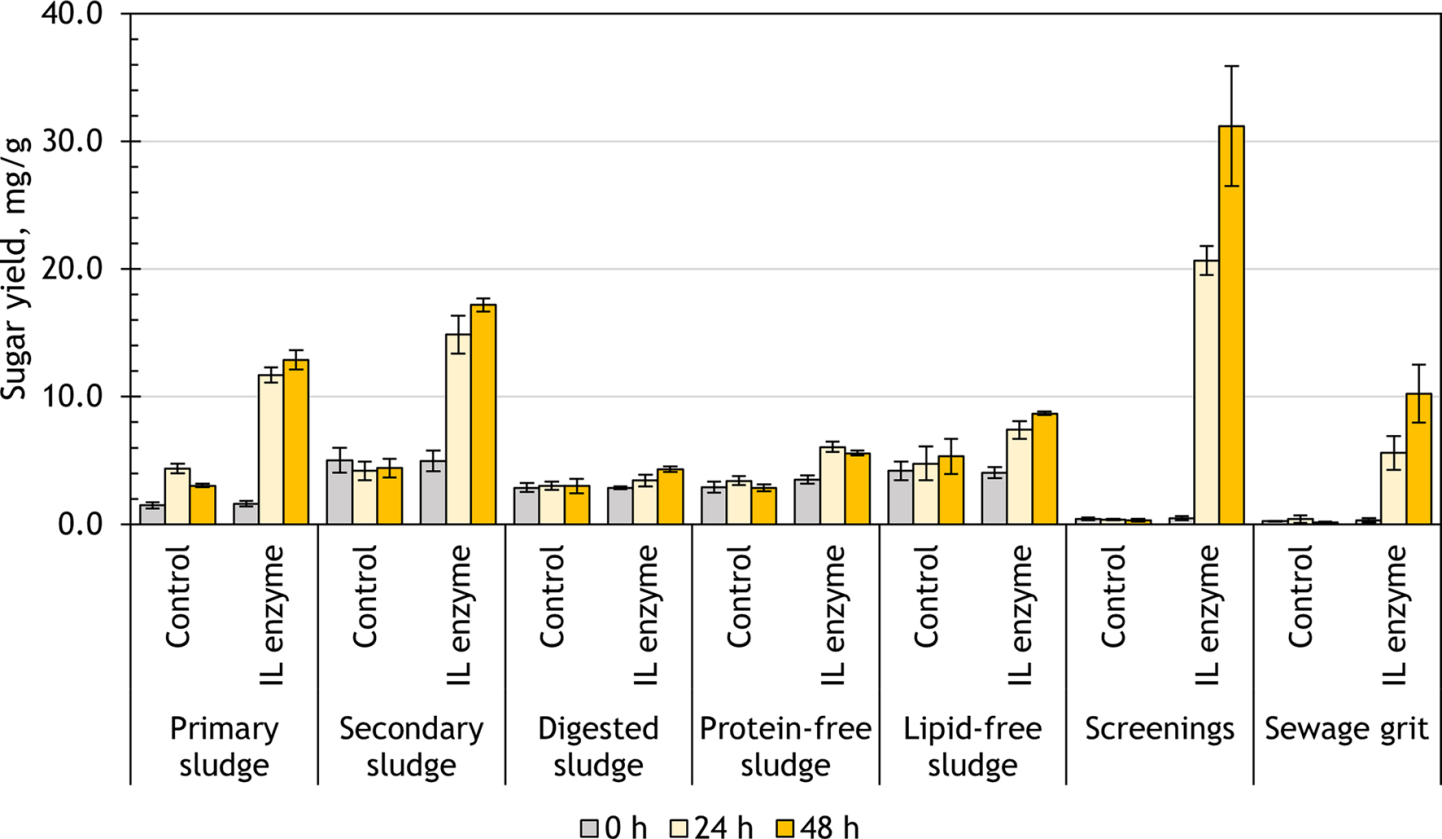
Sugar yield after 48 h of enzymatic hydrolysis with IL enzyme

After 48 h of enzymatic hydrolysis with the laboratory-made IL enzyme, the sugar content increased for all substrates (*p* < 0.05). The highest sugar yield was found in the hydrolysate of screenings (31.2 ± 4.7 mg/g), followed by secondary sludge (17.2 ± 0.5 mg/g), primary sludge (12.8 ± 0.8 mg/g) and sewage grit (10.2 ± 2.3 mg/g) (Fig. 3). The lowest final sugar yield was measured in the hydrolysate of digested sludge (4.3 ± 0.2 mg/g). In the case of the samples that were subjected to resource recovery, the hydrolysate of protein-free sludge contained 5.6 ± 0.2 mg/g sugars. Lipid-free sludge yielded 8.7 ± 0.2 mg/g sugars.

There were no significant differences between the sugar yields at 24 and 48 h for screenings, primary, secondary, protein-free and lipid-free sludge. This finding indicates that the hydrolysis outcome was already achieved at 24 h, with the practical implication that the treatment could be shortened to 24 h but have the same results as the 48 h treatment. For digested sludge and sewage grit, the sugar yield was still changing between 24 and 48 h marks. A longer hydrolysis time or a higher enzyme loading might be needed to reach the optimal sugar yield for these substrates.

**Fig. 4:**
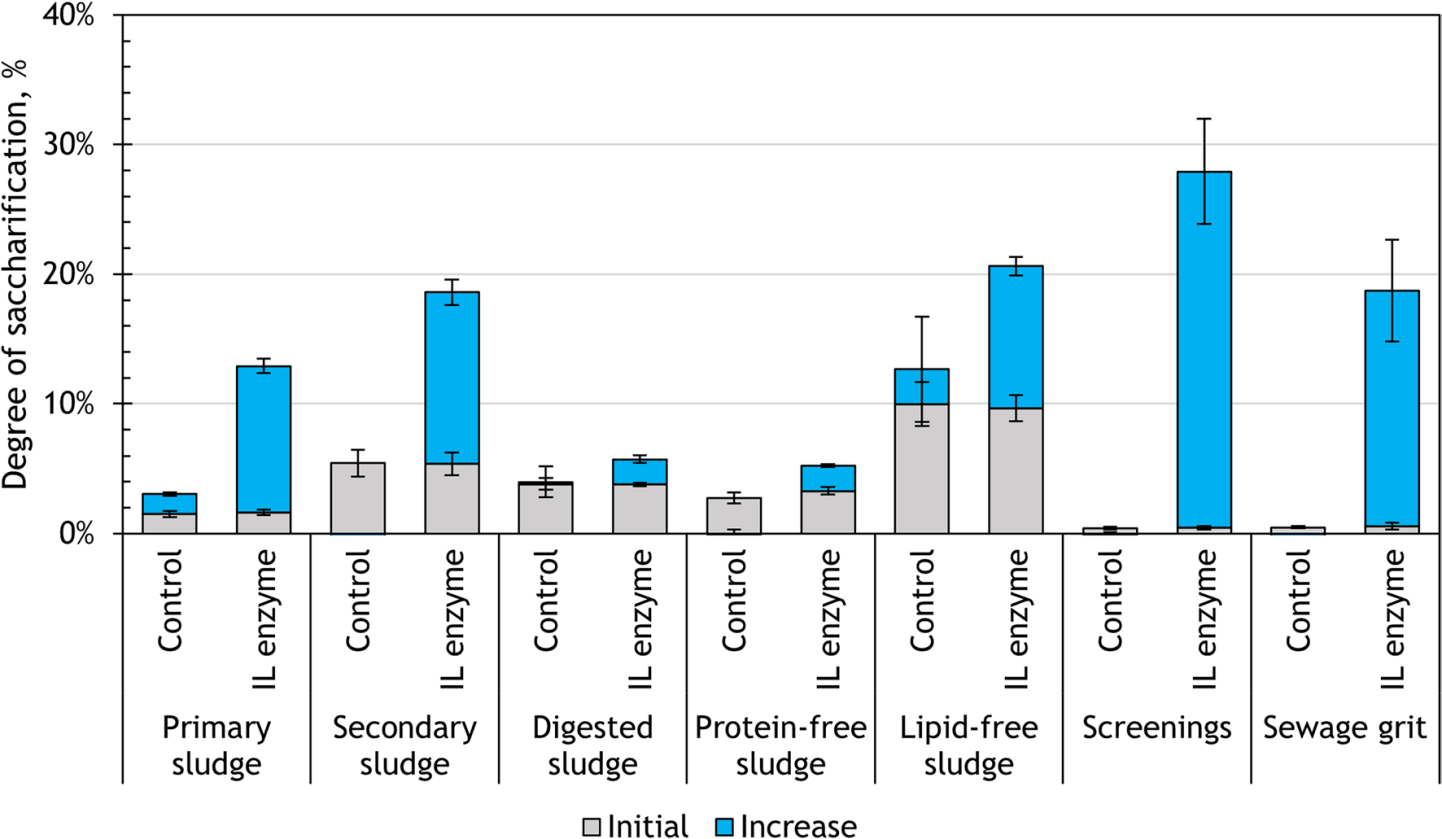
Changes in the degree of saccharification after 48 h of enzymatic hydrolysis with IL enzyme

After 48 h of IL enzyme application, the highest DS was achieved for screenings (28%), followed by lipid-free sludge (21%), sewage grit (19%), and secondary sludge (19%) (Fig. 4). However, each substrate had a different amount of sugars present in the initial hydrolysate (at 0 h), therefore, the hydrolytic activity of the enzymes was additionally described by the observed changes in the DS (stacked bars in Fig. 4). The highest increase in the DS by IL enzyme was observed for screenings (+ 27%), sewage grit (+ 18%), secondary sludge (+ 13%) and primary sludge (+ 11%). The changes in the DS over time are attributed to the activity of the IL enzyme. In the case of lipid-free sludge, although the final DS achieved was among the highest values, its initial hydrolysate already had a DS of 10% and it gained just 11% more over the course of the hydrolysis. Digested sludge and protein-free sludge had the smallest increase in DS (+ 4% and + 2%, respectively).

Overall, the sugar yields after the treatment with IL enzyme were in good agreement with the carbohydrate content in the substrates. Screenings, primary sludge and secondary sludge were the most successful substrates under the IL enzyme treatment.

## Discussion

Compared to the established lignocellulosic feedstocks, wastewater related wastes are less explored as substrates for sugar production, and the use of IL enzymes on them has not been described before. The maximum sugar yield released by primary, secondary, and digested sludge in this study was 13, 17 and 4 mg/g, respectively, while screenings produced a maximum of 101 mg/g. Among these four substrates that were tested with both enzymes, all except screenings achieved their maximums during treatment with IL enzyme. Furthermore, the sugar levels were raised for all substrates during the hydrolysis with IL enzyme. This shows the versatility of the fungal IL enzyme and highlights its possible application beyond the typical lignocellulosic biomass. Overall, the sugar yields produced by IL and commercial enzymes were lower than reported in the literature, except for screenings which achieved 90% saccharification when treated with CC enzyme. It should be noted that the enzymatic hydrolysis of screenings in this study was performed at low temperature conditions (30 °C), at a relatively low enzyme loading rate and without extensive pretreatment, yet it was able to increase the sugar yield of the substrate. The outcome of the enzymatic hydrolysis could be optimized in various ways, such as using a higher enzyme loading, combining enzymes, increasing the temperature, producing the enzymes through different carbon sources and pretreating the samples [51, 52]. Moreover, the IL enzyme employed in this study was a crude mixture, in contrast to the purified commercial enzyme. Purification of the IL enzyme could further improve its performance.

In a recent study of enzymatic hydrolysis with IL enzyme, the hydrolysate of hay contained at most 215 mg/g sugars, while sawdust yielded only 28 mg/g sugars after 48 h [38]. In a study with the same hydrolysis conditions, the sugar yield was 173 mg/g for hay and under 50 mg/g for straw [53]. In this study, the highest sugar yield obtained by IL enzyme was 31 mg/g (screenings), which is in the same range as sawdust and straw.

In another study, primary, secondary and digested sludge were treated with a commercial cellulase for 24 h and produced a maximum of 311 mg/g, 129 mg/g and 11 mg/g sugars, respectively [32]. This result was achieved without any pretreatment for all three types of sludge. Compared to our samples, their sludge contained similar levels of protein, while the content of cellulose and fats was higher. The abundance of carbohydrates was likely a contributing factor to the respectable hydrolysis yield. Their findings show the potential of primary sludge as a bioethanol feedstock, as well as reinforce the poor performance of digested sludge.

Recently, unprocessed screenings were treated with a commercial cellulase and yielded 290 mg/g sugars (86% of the theoretical yield) after 72 h [34]. Additional hydrothermal pretreatment was performed on the screenings but the resulting sugar yield in that case was lower, likely because pretreatment mostly affects the hemicellulose and lignin fractions instead of cellulose. The effect of CC enzyme on screenings in this study (90% saccharification) was achieved without pretreatment (other than drying and grinding) at a lower temperature (30 °C instead of 50 °C) and a shorter time, suggesting that a less energy-intensive process is possible for the valorization of screenings, which would be especially appealing for WWTPs.

The differences in the hydrolytic activity of the enzyme preparations are related to their enzymatic profile. Both IL and CC enzymes contain cellulases and hemicellulases [51, 54, 55]. Additionally, beta-glucosidase is included in the CC enzyme formulation whereas in the IL enzyme it is present at a low activity. Beta-glucosidase is able to hydrolyse cellobiose, an intermediate product of cellulose hydrolysis that inhibits the activity of cellulase [56]. The addition of beta-glucosidase prevents the accumulation of inhibitory products and improves the hydrolysis process. Moreover, IL enzyme cocktail included enzymes responsible for lignin degradation such as laccase, lignin peroxidase and manganese peroxidase [38, 57]. The diverse enzymatic profile of IL might explain its performance and ability to release sugars from such diverse substrates as primary and secondary sludge. Meanwhile, CC was the preferred enzyme for the hydrolysis of screenings. The initial reducing sugar content of screenings was very low (0–1 mg/g), indicating that the carbohydrates in this substrate were complex polymers such as cellulose and thus there was a high compatibility with the cellulase-based CC enzyme.

The growth substrate used for enzyme production affects the enzymatic profile of the preparation and thus its performance [36, 51]. The use of hay as a growth substrate for IL enzyme production may explain its satisfactory effect on agricultural wastes [38, 53] but a poorer performance on sewage sludge (this study). One of the advantages of producing enzymes in a laboratory is the possibility of selecting the carbon source which is used for enzyme production. For example, *I. lacteus* produced beta-glucosidase, xylanase, endoglucanase and exoglucanase when agricultural digestate was used as the carbon source [36], while the production of lignin peroxidase by *Phanerochaete chrysosporium* was observed when using secondary sludge as the carbon source [58]. Another study described the production of a cellulase enzyme blend by *Trichoderma harzianum* using wastewater as the culture medium [44]. For improved production of fungal enzymes that are suited for the hydrolysis of WWTP wastes, we suggest using a microorganism isolated from sewage sludge or screenings and the same material as a carbon source during enzyme production.

It could be worthwhile to explore the combination of IL enzyme and commercial cellulases for improved outcomes of enzymatic hydrolysis, as considered by Supaporn et al. (2022) [27] due to the improved synergy between enzymes. A consortium of enzymes is likely to achieve a higher sugar yield even at a low enzyme loading, compared to individual cellulases [59]. Optimizing the enzymatic profile is likely a more efficient strategy for improving the sugar yields than increasing the enzyme loading [60].

Furthermore, the selection of the enzyme-producing microorganism is another variable available for optimization. In the case of sewage sludge, it could be worthwhile to explore the cellulolytic potential of the native microorganisms. As demonstrated by Prasetyo et al. (2011) [21], a cellulase producer was successfully isolated from paper sludge which performed at the same level as a commercial enzyme, releasing 130 mg/g sugars from paper sludge.

A major roadblock for the success of enzymatic hydrolysis can be the presence of inhibitors that block the action of cellulolytic enzymes [60, 61]. Inhibitors include compounds such as phenols, organic acids, furan aldehydes, and inorganic ions, as well as accumulated products like cellobiose and glucose [62]. Another concern is the presence of humic substances which are generated as part of organic matter degradation during wastewater treatment and are also released during the anaerobic digestion of sludge [63]. Humic substances are prone to binding to organic compounds such as amino acids in enzymes. The interaction of humic substances in sewage sludge with the enzyme preparations could explain the poor hydrolytic effect observed with the sludge substrates, particularly with digested sludge.

Lipid-free and protein-free sludge were prepared and subjected to enzymatic hydrolysis with IL enzyme to determine the impact of resource recovery on the sugar production. Both substrates released less sugars than their precursors (primary and secondary sludge) as well as less than the other substrates. This is likely attributed to the sample processing, for example, washing and ultrasonication steps, which were not highly selective and may have removed carbohydrates from the samples along with the target resources, thus reducing the amount of the material available for saccharification. Similar to the lipid-free sludge in this study, Supaporn et al. (2018) produced lipid-extracted sludge from sewage sludge by chloroform–methanol extraction. It contained 2.6% lipids while the starting material had 14.5% lipids [28]. Their extraction method retained all carbohydrates (8.9%), while in our preparation of lipid-free sludge, the carbohydrates were partially lost. The maximum sugar yield for lipid-extracted sludge was 76.3% [27], showing the benefits of integrating resource recovery with a sugar production workflow.

Our results show that the presence of lipids and proteins did not inhibit the enzymatic hydrolysis of primary and secondary sludge, and that the removal of these fractions decreased the sugar yields. Based on the results, it can be concluded that removing proteins and lipids prior to the hydrolysis with IL enzyme was not advantageous. The integration of resource recovery and enzymatic hydrolysis should pay attention to the compatibility of parameters such as pH, trace organic solvents and the presence of hydrolysis inhibitors. It could be beneficial to conduct the treatment in a reversed order, i.e., starting with the enzymatic hydrolysis of sludge and continuing with resource recovery on the hydrolysed biomass residues.

The introduction of sugar production at WWTPs will have broad environmental implications. This technology could become a source of local renewable energy, covering a part of the energy demands of a WWTP, especially in situations where land application of sludge is not possible. The reduction of the amount of landfilled waste in the case of screenings would be another environmental benefit. Overall, the recycling of waste streams fits in very well with the principles of circular economy and strengthens the connection between waste, wastewater, and energy sectors.

## Conclusions

Enzymatic hydrolysis was performed at low temperature conditions on various substrates originating from wastewater treatment plants, such as primary sludge, secondary sludge and screenings, to evaluate their potential for sugar production. After 48 h hydrolysis with IL enzyme at 4 FPU/g, screenings reached the highest sugar yield (31.2 mg/g) and the highest saccharification (28%). For primary sludge, the best outcome was 12.8 mg/g sugars (13% DS). The application of IL enzyme resulted in the release of sugars from all sewage-related substrates, demonstrating the versatility of this enzyme source. The highest saccharification by CC enzyme was achieved during the hydrolysis of screenings (101 mg/g, 90%), highlighting the valorization potential of this waste stream that is currently destined for landfill. The highest observed increase in the degree of saccharification of digested sludge was just 2%, demonstrating its unsuitability as a feedstock for sugar production. Protein and lipid recovery from sewage sludge prior to the enzymatic hydrolysis did not improve the sugar yield, showing the challenges of integrating resource recovery and saccharification processes. Improvements in sample pretreatment, fungal enzyme preparation and hydrolysis conditions should unlock the path towards efficient bioethanol production and the transition of WWTPs into water resource recovery facilities.

## Data Availability

The datasets used and/or analysed during the current study are available from the corresponding author on reasonable request.
